# Traditional Medicines in Africa: An Appraisal of Ten Potent African Medicinal Plants

**DOI:** 10.1155/2013/617459

**Published:** 2013-12-03

**Authors:** M. Fawzi Mahomoodally

**Affiliations:** Department of Health Sciences, Faculty of Science, University of Mauritius, 230 Réduit, Mauritius

## Abstract

The use of medicinal plants as a fundamental component of the African traditional healthcare system is perhaps the oldest and the most assorted of all therapeutic systems. In many parts of rural Africa, traditional healers prescribing medicinal plants are the most easily accessible and affordable health resource available to the local community and at times the only therapy that subsists. Nonetheless, there is still a paucity of updated comprehensive compilation of promising medicinal plants from the African continent. The major focus of the present review is to provide an updated overview of 10 promising medicinal plants from the African biodiversity which have short- as well as long-term potential to be developed as future phytopharmaceuticals to treat and/or manage panoply of infectious and chronic conditions. In this endeavour, key scientific databases have been probed to investigate trends in the rapidly increasing number of scientific publications on African traditional medicinal plants. Within the framework of enhancing the significance of traditional African medicinal plants, aspects such as traditional use, phytochemical profile, *in vitro*, *in vivo*, and clinical studies and also future challenges pertaining to the use of these plants have been explored.

## 1. Introduction

Traditional medicine is the sum total of knowledge, skills, and practices based on the theories, beliefs, and experiences indigenous to different cultures that are used to maintain health, as well as to prevent, diagnose, improve, or treat physical and mental illnesses [1]. Traditional medicine that has been adopted by other populations (outside its indigenous culture) is often termed complementary or alternative medicine (CAM) [1, 2].

The World Health Organization (WHO) reported that 80% of the emerging world's population relies on traditional medicine for therapy. During the past decades, the developed world has also witnessed an ascending trend in the utilization of CAM, particularly herbal remedies [3]. Herbal medicines include herbs, herbal materials, herbal preparations, and finished herbal products that contain parts of plants or other plant materials as active ingredients. While 90% of the population in Ethiopia use herbal remedies for their primary healthcare, surveys carried out in developed countries like Germany and Canada tend to show that at least 70% of their population have tried CAM at least once [2, 3]. It is likely that the profound knowledge of herbal remedies in traditional cultures, developed through trial and error over many centuries, along with the most important cures was carefully passed on verbally from one generation to another. Indeed, modern allopathic medicine has its roots in this ancient medicine, and it is likely that many important new remedies will be developed and commercialized in the future from the African biodiversity, as it has been till now, by following the leads provided by traditional knowledge and experiences [2–5].

The extensive use of traditional medicine in Africa, composed mainly of medicinal plants, has been argued to be linked to cultural and economic reasons. This is why the WHO encourages African member states to promote and integrate traditional medical practices in their health system [1]. Plants typically contain mixtures of different phytochemicals, also known as secondary metabolites that may act individually, additively, or in synergy to improve health. Indeed, medicinal plants, unlike pharmacological drugs, commonly have several chemicals working together catalytically and synergistically to produce a combined effect that surpasses the total activity of the individual constituents. The combined actions of these substances tend to increase the activity of the main medicinal constituent by speeding up or slowing down its assimilation in the body. Secondary metabolites from plant's origins might increase the stability of the active compound(s) or phytochemicals, minimize the rate of undesired adverse side effects, and have an additive, potentiating, or antagonistic effect. It has been postulated that the enormous diversity of chemical structures found in these plants is not waste products, but specialized secondary metabolites involved in the relationship of the organism with the environment, for example, attractants of pollinators, signal products, defensive substances against predators and parasites, or in resistance against pests and diseases. A single plant may, for example, contain bitter substances that stimulate digestion and possess anti-inflammatory compounds that reduce swellings and pain, phenolic compounds that can act as an antioxidant and venotonics, antibacterial and antifungal tannins that act as natural antibiotics, diuretic substances that enhance the elimination of waste products and toxins, and alkaloids that enhance mood and give a sense of well-being [1–5]. Although some may view the isolation of phytochemicals and their use as single chemical entities as a better alternative and which have resulted in the replacement of plant extracts' use, nowadays, a view that there may be some advantages of the medical use of crude and/or standardized extracts as opposed to isolated single compound is gaining much momentum in the scientific community.

## 2. African Traditional Medicine

African traditional medicine is the oldest, and perhaps the most assorted, of all therapeutic systems. Africa is considered to be the cradle of mankind with a rich biological and cultural diversity marked by regional differences in healing practices [2, 6]. African traditional medicine in its varied forms is holistic involving both the body and the mind. The traditional healer typically diagnoses and treats the psychological basis of an illness before prescribing medicines, particularly medicinal plants to treat the symptoms [2, 6–8]. The sustained interest in traditional medicine in the African healthcare system can be justified by two major reasons. The first one is inadequate access to allopathic medicines and western forms of treatments, whereby the majority of people in Africa cannot afford access to modern medical care either because it is too costly or because there are no medical service providers. Second, there is a lack of effective modern medical treatment for some ailments such as malaria and/or HIV/AIDS, which, although global in distribution, disproportionately affect Africa more than other areas in the world.

The most common traditional medicine in common practice across the African continent is the use of medicinal plants. In many parts of Africa, medicinal plants are the most easily accessible health resource available to the community. In addition, they are most often the preferred option for the patients. For most of these people, traditional healers offer information, counseling, and treatment to patients and their families in a personal manner as well as having an understanding of their patient's environment [2, 6, 7]. Indeed, Africa is blessed with enormous biodiversity resources and it is estimated to contain between 40 and 45,000 species of plant with a potential for development and out of which 5,000 species are used medicinally. This is not surprising since Africa is located within the tropical and subtropical climate and it is a known fact that plants accumulate important secondary metabolites through evolution as a natural means of surviving in a hostile environment [9]. Because of her tropical conditions, Africa has an unfair share of strong ultraviolet rays of the tropical sunlight and numerous pathogenic microbes, including several species of bacteria, fungi, and viruses, suggesting that African plants could accumulate chemopreventive substances more than plants from the northern hemisphere. Interestingly, Abegaz et al. [10] have observed that of all species of *Dorstenia* (Moraceae) analysed, only the African species, *Dorstenia mannii* Hook.f, a perennial herb growing in the tropical rain forest of Central Africa contained more biological activity than related species [9–11].

Nonetheless, the documentation of medicinal uses of African plants and traditional systems is becoming a pressing need because of the rapid loss of the natural habitats of some of these plants due to anthropogenic activities and also due to an erosion of valuable traditional knowledge. It has been reported that Africa has some 216 million hectares of forest, but the African continent is also notorious to have one of the highest rates of deforestation in the world, with a calculated loss through deforestation of 1% per annum [7, 12]. Interestingly, the continent also has the highest rate of endemism, with the Republic of Madagascar topping the list by 82%, and it is worth to emphasize that Africa already contributes nearly 25% of the world trade in biodiversity. Nonetheless, the paradox is that in spite of this huge potential and diversity, the African continent has only few drugs commercialized globally [2, 12, 13].

The scientific literature has witnessed a growing number of publications geared towards evaluating the efficacy of medicinal plants from Africa which are believed to have an important contribution in the maintenance of health and in the introduction of new treatments. Nonetheless, there is still a dearth of updated comprehensive compilation of promising medicinal plants from the African continent.

The main aim of the present review is to highlight the importance and potential of medicinal plants from the African biodiversity which have short- as well as long-term potential to be developed as future phytopharmaceuticals to treat and/or manage panoply of infectious and chronic conditions. The review might also provide a starting point for future studies aimed at isolation, purification, and characterization of bioactive compounds present in these plants as well as exploring the potential niche market of these plants. In this endeavor, major scientific databases such as EBSCOhost, PubMed Central, Scopus (Elsevier), and Emerald amongst others have been probed to investigate trends in the rapidly increasing number of scientific publications on African traditional medicinal plants. Ten medicinal plants (*Acacia senegal*, *Aloe ferox*, *Artemisia herba-alba*, *Aspalathus linearis*, *Centella asiatica*, *Catharanthus roseus*, *Cyclopia genistoides*, *Harpagophytum procumbens*, *Momordica charantia*, and *Pelargonium sidoides*) of special interest were chosen for more detailed reviews based on the following criteria: medicinal plants that form part of African herbal pharmacopeia with commercial importance and those plants from which modern phytopharmaceuticals have been derived.

### 2.1. **Acacia senegal** (L.) Willd. (Leguminosae: Mimosoideae)—Gum Arabic

*Acacia senegal*, also known as gum Arabic, is native to semidesert and drier regions of sub-Saharan Africa, but widespread from Southern to Northern Africa. It is used as a medicinal plant in parts of Northern Nigeria, West Africa, North Africa, and other parts of the world [8]. The use of gum arabic (or gum acacia), which is derived from an exudate from the bark, dates from the first Egyptian Dynasty (3400 B.C.). It was used in the production of ink, which was made from a mixture of carbon, gum, and water. Inscriptions from the 18th Dynasty refer to this gum as “komi” or “komme.” Gum arabic has been used for at least 4,000 years by local people for the preparation of food, in human and veterinary medicine, in crafts, and as a cosmetic. The gum of *A. senegal* has been used medicinally for centuries, and various parts of the plant are used to treat infections such as bleeding, bronchitis, diarrhea, gonorrhea, leprosy, typhoid fever, and upper respiratory tract infections. African herbalists use gum acacia to bind pills and to stabilize emulsions. It is also used in aromatherapy for applying essential oils [8, 14–16].

Currently,* A. senegal* is an important naturally occurring oil-in-water emulsifier, which is in regular use in the food and pharmaceutical industries. Medicinally, gum arabic is used extensively in pharmaceutical preparations and is a food additive approved as toxicologically safe by the Codex Alimentarius. It has been used as demulcent, skin protective agent, and pharmaceutical aids such as emulsifier and stabilizer of suspensions and additives for solid formulations. It is sometimes used to treat bacterial and fungal infections of the skin and mouth. It has been reported to soothe the mucous membranes of the intestines and to treat inflamed skin [17, 18]. The demulcent, emollient gum is used internally against inflammation of intestinal mucosa and externally to cover inflamed surfaces, as burns, sore nipples, and nodular leprosy. Additionally, it has also been documented to be used as antitussive, expectorant, astringent, catarrh and against colds, coughs, diarrhea, dysentery, gonorrhea, hemorrhage, sore throat, typhoid, and for urinary tract ailments [18]. The gum of *A. senegal* has been pharmaceutically used mainly in the manufacture of emulsions and in making pills and troches (as an excipient), as demulcent for inflammations of the throat or stomach and as masking agent for acrid tasting substances such as capsicum and also as a film-forming agent in peel-off masks. Gum arabic is also used widely as an ingredient in foods like candies and soft drinks as the gum has the properties of glue that is safe to eat. Gum acacia is widely used in organic products as natural alternative to chemical binders and is used in commercial emulsification for the production of beverages and flavor concentrates [8, 17–19].

Recently, it has been reported that *A. senegal* bark extracts were evaluated *in vitro* for their antimicrobial potential against human pathogenic isolates (*Escherichia coli*, *Staphylococcus aureus*, *Streptococcus pneumoniae*, *Klebsiella pneumoniae*, *Shigella dysenteriae*, *Salmonella typhi*, *Streptococcus pyogenes*, *Pseudomonas aeruginosa*, and *Proteus vulgaris*). The extract was found to exhibit significant antibacterial activity which was suggested to be due to the presence of tannins and saponins in the plant. It was also reported that the plant extract may not be toxic to man following *in vitro* cytotoxicity evaluation [18].

### 2.2. **Aloe ferox** Mill. (Xanthorrhoeaceae)—Bitter Aloe or Cape Aloe

*Aloe ferox* is native to South Africa and Lesotho and is considered to be the most common Aloe species in South Africa. *A. ferox* has been used since time immemorial and has a well-documented history of use as an alternative medicine and is one of the few plants depicted in San rock paintings. The bitter latex, known as Cape aloe, is used as laxative medicine in Africa and Europe and is considered to have bitter tonic, antioxidant, anti-inflammatory, antimicrobial, and anticancer properties [8, 19–23].

The use of *A. ferox* as a multipurpose traditional medicine has been translated into several commercial applications and it is a highly valued plant in the pharmaceutical, natural health, food, and cosmetic industries. *A. ferox* is considered South Africa's main wild harvested commercially traded species. The finished product obtained from aloe tapping, aloe bitters, has remained a key South African export product since 1761 when it was first exported to Europe. The aloe tapping industry is the livelihood of many rural communities and formalization of the industry in the form of establishment of cooperatives and trade agreements. It has been suggested that its trade may have an extensive poverty alleviation effect in Africa [19, 24, 25].

*A. ferox* has many traditional and documented medicinal uses. It is most popularly used for its laxative effect and as a topical application to the skin, eyes, and mucous membranes. Scientific studies conducted have verified many of the traditional uses. More recently, the cosmetic industry has shown interest in *A. ferox* gel [8, 19]. It has been reported that *A. ferox *gel contains at least 130 medicinal agents with anti-inflammatory, analgesic, calming, antiseptic, germicidal, antiviral, antiparasitic, antitumour, and anticancer effects encompassing all of the traditional uses and scientific studies done on *A. ferox* and its constituents [8, 24–31].

A wide variety of phenolic compounds (chromones, anthraquinones, anthrone, anthrone-C-glycosides, and other phenolic compounds) are present within *A. ferox* and these compounds have been well documented to possess biological activity [21, 24–31]. The gel polysaccharides are known to be of the arabinogalactan and rhamnogalacturonan types. The leaf gel composition still remains unknown and its claimed biological activities still remain to be investigated. The active ingredient (purgative principle) is a chemical compound known as Aloin (also called Barbaloin) [19]. The gel has also been found to be rich in antioxidant polyphenols, indoles, and alkaloids. Tests carried out have shown that the nonflavonoid polyphenols contribute to the majority of the total polyphenol content. With this phytochemical profile, *A. ferox* leaf gel has been identified to be very promising in alleviating symptoms associated with/or prevention of common noncommunicable diseases such as cardiovascular diseases, cancer, neurodegeneration, and diabetes [32].

The leaves have been reported to contain two juices; the yellow bitter sap is used as laxative while the white aloe gel is used in health drinks and skin care products. This purgative drug is used for stomach complaints, mainly as a laxative to “purify” the stomach and also as a bitter tonic (*amarum*) in various digestives and stomachics (such as “Lewensessens” and “Swedish Bitters”). Usually, a small crystal of the drug (0.05–0.2 g) is taken orally as a laxative. Half the laxative dose is suggested for arthritis. The fresh bitter sap is instilled directly against conjunctivitis and sinusitis [24, 25]. It is well known that bitter substances stimulate the flow of gastric juices and in so doing improve digestion. The fresh juice emanating from the cut leaf is also applied on burn wounds [33]. *A. ferox* is claimed to detoxify the damaged surface area and exhibit analgesic and anesthetic properties while promoting new tissue formation (granulation) which fills the wound. It was demonstrated that *A. ferox* enriched with aloins can inhibit collagenase and metalloprotease activity, which can degrade collagen connective tissues. The effect of *A. ferox* whole leaf juice on wound healing and skin repair was investigated in an animal model and its safety was evaluated. The results showed that the *A. ferox* whole leaf juice preparation accelerates wound closure and selectively inhibits microbial growth. No dermal toxicity or side effects were observed during the experimental period [23].

### 2.3. **Artemisia herba**-**alba ** Asso (Med)—Asteraceae—Wormwood

*Artemisia herba*-*alba* is commonly known as wormwood or desert wormwood (known in Arabic as shih, and as Armoise blanche in French). It is a greyish strongly aromatic perennial dwarf shrub native to the Northern Africa, Arabian Peninsula, and Western Asia [34]. *A. herba*-*alba* has been used in folk medicine by many cultures since ancient times. In Moroccan folk medicine, it is used to treat arterial hypertension and diabetes and in Tunisia, it is used to treat diabetes, bronchitis, diarrhea, hypertension, and neuralgias [34, 35]. Herbal tea from *A. herba*-*alba* has been used as analgesic, antibacterial, antispasmodic, and hemostatic agents in folk medicines [34–39]. During an ethnopharmacological survey carried out among the Bedouins of the Negev desert, it was found that *A. herba*-*alba* was used to mitigate stomach disorders. This plant is also suggested to be important as a fodder for sheep and for livestock in the plateau regions of Algeria where it grows abundantly. It has also been reported that Ascaridae from hogs and ground worms were killed by the oil of the Libyan *A. herba*-*alba* in a short time [34–42].

Oral administration of 0.39 g/kg body weight of the aqueous extract of the leaves or barks of *A. herba*-*alba* has been documented to produce a significant reduction in blood glucose level, while the aqueous extract of roots and methanolic extract of the aerial parts of the plant produce almost no reduction in blood glucose level. The extract of the aerial parts of the plant seems to have minimal adverse effect and high LD_50_ value [19, 30].

Among *A. herba*-*alba* phytochemical constituents, essential oils have been extensively studied, with several chemotypes being recognized. The variability from the essential oils isolated from *A. herba*-*alba* collected in Algeria, Israel, Morocco, and Spain was revised by Dob and Benabdelkader [43, 44], but, since then, many other studies have reinforced its high chemical polymorphism. Recently, fifty components were identified in *A. herba*-*alba* oils, oxygen-containing monoterpenes being dominant in all cases (72–80%). Camphor (17–33%), *α*-thujone (7–28%), and chrysanthenone (4–19%) were the major oil components. Despite the similarity in main components, three types of oils could be defined: (a) *α*-thujone : camphor (23–28 : 17–28%), (b) camphor : chrysanthenone (33 : 12%), and (c) *α*-thujone : camphor : chrysanthenone (24 : 19 : 19%) [43].

The antifungal activity of *Artemisia herba*-*alba* was found to be associated with two major volatile compounds isolated from the fresh leaves of the plant. Carvone and piperitone were isolated from *Artemisia herba*-*alba*. The antifungal activity of the purified compounds carvone and piperitone was estimated to be 5 *μ*g/mL and 2 *μ*g/mL against *Penicillium citrinum* and 7 *μ*g/mL and 1.5 *μ*g/mL against *Mucor rouxii*, respectively. In another study, the antifungal activity of the constituents and biological activities of *Artemisia herba*-*alba* essential oils of 25 Moroccan medicinal plants, including *A. herba*-*alba*, against *Penicillium digitatum*, *Phytophthora citrophthora*, *Geotrichum citri*-*aurantii*, and *Botrytis cinerea* have been reported [42, 43].

### 2.4. **Aspalathus linearis** (Brum.f.) R. Dahlg. (Fabaceae)—Rooibos

*Aspalathus linearis*, an endemic South African fynbos species, is cultivated to produce the well-known herbal tea, also commonly known as rooibos. Its caffeine-free and comparatively low tannin status, combined with its potential health-promoting properties, most notably antioxidant activity, has contributed to its popularity and consumer acceptance globally. The utilization of rooibos has also moved beyond a herbal tea to intermediate value-added products such as extracts for the beverage, food, nutraceuticals and cosmetic markets [45–52].

Rooibos is used traditionally throughout Africa in numerous ways. It has been used as a refreshment drink and as a healthy tea beverage [8, 19]. It was only after the discovery that an infusion of rooibos, when administered to her colicky baby, cured the chronic restlessness, vomiting, and stomach cramps that rooibos became well known as a “healthy” beverage, leading to a broader consumer base. Many babies since then have been nurtured with rooibos—either added to their milk or given as a weak brew [8, 16, 45–52].

Animal studies have suggested that it has potent antioxidant, immunomodulating, and chemopreventive effects. The plant is rich in minerals and low in tannins. Among the flavonoids present are the unique C-glucoside dihydrochalcones: aspalathin and nothofagin and with aspalathin being the most abundant. *In vitro* data has shown that the daily intake of the alkaline extracts of the red rooibos tea could suppress HIV infections in the extract, though clinical evaluation has yet to be conducted [8, 45]. There is growing evidence that the flavonoids present in the plant contribute substantially to a reduction in cardiovascular disease and other ailments associated with ageing. Recent studies have shown that aspalathin has beneficial effects on glucose homeostasis in Type 2 diabetes through stimulating glucose uptake in muscle tissues and insulin secretion from pancreatic beta-cells [8, 48]. The unfermented rooibos has been found to have greater chemoprotective effects than the fermented variety [49]. Aspalathin has free-radical capturing properties and is absorbed through the small intestine as such [50].

The bronchodilator, antispasmodic, and blood pressure lowering effects of rooibos tea have been confirmed *in vitro* and *in vivo*. It has also been reported that the antispasmodic effect of the rooibos is mediated predominantly through potassium ionchannel activation [51, 52]. There is also increasing evidence of antimutagenic effects. Animal study suggested the prevention of age-related accumulation of lipid peroxidases in the brain [19, 26, 47].

Rooibos is becoming more popular in western countries particularly among health-conscious consumers, due to the absence of alkaloids and low tannin content. It is also reported to have a high level of antioxidants such as aspalathin and nothofagin. The antioxidative effect has also been attributed to the presence of water-soluble polyphenols such as rutin and quercetin [53]. Rooibos is purported to assist nervous tension, allergies, and digestive problems.

Rooibos extracts, usually combined with other ingredients, are available in pill form, but these products fall in the category of dietary supplements. Recent research has underscored the potential of aspalathin and selected rooibos extracts such as an aspalathin-enriched green rooibos extract as antidiabetic agents [54–68]. A patent application for the use of aspalathin in this context was filed in Japan, while a placebo-controlled trial application was filed for the use of rooibos extract as an antidiabetic agent [66]. It is claimed that rooibos extract and a hetero-dimer containing aspalathin, isolated from rooibos, could be used as a drug for the treatment of neurological and psychiatric disorders of the central nervous system [19, 68]. Other opportunities may lie with topical skin products. Two studies concerning inhibition of COX-2 in mouse skin [67] and inhibition of mouse skin tumor promotion tend to support the role of the topical application of rooibos extract. Nonetheless, more research would be needed to explore its potential in preventing skin cancer in humans [67].

### 2.5. **Centella asiatica** (L.) Urb. (Apiaceae)—Centella

*Centella asiatica* is a medicinal plant that has been used since prehistoric times. It has a pan-tropical distribution and is used in many healing cultures, including Ayurvedic medicine, Chinese traditional medicine, Kampo (Japanese traditional medicine), and African traditional medicine [19, 69]. To date, it continues to be used within the structure of folk medicine and is increasingly being located at the interface between traditional and modern scientifically oriented medicine. Traditionally, *C. asiatica* is used mainly for wound healing, burns, ulcers, leprosy, tuberculosis, lupus, skin diseases, eye diseases, fever, inflammation, asthma, hypertension, rheumatism, syphilis, epilepsy, diarrhea, and mental illness and is also eaten as a vegetable or used as a spice. In Mauritius, the application of *C. asiatica* in the treatment of leprosy was reported for the first time in 1852 while the clinical use of *C. asiatica*, as a therapeutic agent suitable for the treatment of leprous lesions, has been documented since 1887 [19].

The active constituents are characterized by their clinical effects in the treatment of chronic venous disease, wound healing, and cognitive functions amongst others [19]. *C. asiatica* contains a variety of pentacyclic triterpenoids that have been extensively studied. Asiaticoside and madecassoside are the two most important active compounds that are used in drug preparations. Both are commercially used mainly as wound-healing agent, based on their anti-inflammatory effects. One of the main active constituents of *C. asiatica* is the ursane-type triterpene saponin, asiaticoside, which is responsible for wound healing properties [19, 70, 71] and is known to stimulate type 1 collagen synthesis in fibroblast cells [72]. Plants collected from various geographical regions and locations in India, Madagascar, Malaysia, Sri Lanka, Andaman Islands, and South Africa have yielded concentrations of asiaticoside ranging from 0.006 to 6.42% of dry weight [73, 74]. *C. asiatica* also contains several other triterpene saponins. Madecassoside always co-occurs with asiaticoside as a main compound and other saponins have been reported, such as asiaticosides A to G, centelloside, brahmoside, and many others [19, 75]. Madagascar plays a major role in *C. asiatica* trade. It is the first producer of *C. asiatica* products worldwide and due to a higher Asiaticoside content of dried leaves, Malagasy origin is appreciated by industry [9].

The ethyl acetate fraction of *C. asiatica* has been reported to increase the effect of the *i.p.* administrated antiepileptic drugs phenytoin, valproate, and gabapentin [75, 76] and was found to decrease the pentylenetetrazol- (PTZ-) kindled induced seizures in rats [75, 77]. This effect might be due to an increase in gamma-aminobutyric acid (GABA) levels caused by the extract as reported by Chatterjee et al. [78]. The neuroprotective properties of the plant in monosodium glutamate treated rats were investigated by Ramanathan et al. [79]. The general behavior, locomotor activity, and the CA1 region of the hippocampus were protected by *C. asiatica* extracts. The levels of catalase, superoxide dismutase, and lipid peroxidase in the hippocampus and striatum were improved indicating a neuroprotective property of the extract [74]. Additionally, the effect of *C. asiatica* on cognitive function of healthy elderly volunteer was evaluated in a randomized, placebo-controlled, double-blind study involving 28 healthy elderly participants. The subjects have received the plant extract at various doses ranging from 250 to 500 and 750 mg once daily for 2 months, and cognitive performance and mood modulation were assessed. It was found that high dose of the plant extract enhanced working memory and increased N100 component amplitude of event-related potential. Improvements of self-rated mood were also found following the *C. asiatica* treatment. The high dose of *C. asiatica* used in the study was suggested to increase calmness and alertness after 1 and 2 months of treatment. However, the precise mechanism(s) underlying these effects still require further investigation [72].

### 2.6. **Catharanthus roseus** (L.) G. Don (Apocynaceae)—Madagascan Periwinkle

*Catharanthus roseus* (Madagascar periwinkle) is a well-known medicinal plant that has its root from the African continent. The interest in this species arises from its therapeutic role, as it is the source of the anticancer alkaloids vincristine and vinblastine, whose complexity renders them impossible to be synthesized in the laboratory; the leaves of this species are still, today, the only source [8, 12, 19, 80]. *C. roseus* originates from Madagascar but now has a wide distribution throughout the tropics, and the story on the traditional utilisation of this plant can be retraced to Madagascar where healers have been using it extensively to treat panoply of ailments. It is commonly used in traditional medicine as a bitter tonic, galactogogue, and emetic. Application for treatment of rheumatism, skin disorders, and venereal diseases has also been reported [8, 10, 19].

*C. roseus* has been found to contain a plethora of phytochemicals (as many as 130 constituents) and the principal component is vindoline (up to 0.5%). Other biologically active compounds are serpentine, catharanthine, ajmalicine (raubasine), akuammine, lochnerine, lochnericine, and tetrahydroalstonine. The plant is also rich in bisindole alkaloids; vindoline and catharanthine are found in very small amounts: vincristine (=leurocristine) in up to 3 g/t of dried drug and vinblastine (=vincaleucoblastine) in a slightly larger amount [8, 12, 19, 81–84].

The oral administration of water-soluble fractions and ethanolic extracts of the leaves have been found to show significant dose-dependent reduction in the blood sugar at 4 h by 26.2, 31.4, 35.6, and 33.4%, respectively, in normal rats. In addition, oral administration of 500 mg/kg 3.5 h before oral glucose tolerance test (10 mg/kg) and 72 h after STZ administration (50 mg/kg IP) in rats showed significant antihyperglycaemic effects. No gross behavioural changes and toxic effects were observed up to 4 mg/kg IP [85].

Extract at dose of 500 mg/kg given orally for 7 and 15 days showed 48.6 and 57.6% hypoglycemic activity, respectively. Prior treatment at the same dose for 30 days provided complete protection against streptozotocin (STZ) challenge (75 mg/kg/i.p. × 1). Enzymatic activities of glycogen synthase, glucose 6-phosphate-dehydrogenase, succinate dehydrogenase, and malate dehydrogenase were decreased in liver of diabetic animals in comparison to normal ones and were significantly improved after treatment with extract at dose of 500 mg/kg p.o. for 7 days. Results indicate increased metabolization of glucose in treated rats. Increased levels of lipid peroxidation measured as 2-thiobarbituric acid reactive substances indicative of oxidative stress in diabetic rats were also normalized by treatment with the extract [2].

Vincristine and Vinblastine are antimitotics as they bind to tubulin and prevent the formation of microtubules that assist in the formation of the mitotic spindle; in this way, they block mitosis in the metaphase. These alkaloids are highly toxic; they both have neurotoxic activity (especially vincristine) because the microtubule assembly also plays a role in neurotransmission. Their peripheral neurotoxic effects are neuralgia, myalgia, paresthesia, loss of the tendon reflexes, depression, and headache, and their central neurotoxic effects are convulsive episodes and respiratory difficulties. Other side effects are multiple and include alopecia, gastrointestinal distress including constipation, ulcerations of the mouth, amenorrhoea, and azoospermia [10, 19, 80, 84, 85].

Recently, new phenolics in different plant parts (leaves, stems, seeds, and petals), including flavonoids and phenolic acids, were reported [10, 19, 82]. In addition, a phytochemical study concerning several classes of metabolites was performed and bioactivity was assessed [81–84]. The high antioxidant potential of *C. roseus* was demonstrated *in vitro* using the radicals DPPH, superoxide, and nitric oxide [19, 81].

### 2.7. **Cyclopia genistoides** (L.) Vent. (Fabaceae)—Honeybush

*Cyclopia genistoides* is an indigenous herbal tea to South Africa and considered as a health food. Traditionally, the leafy shoots and flowers were fermented and dried to prepare tea. It has also been used since early times for its direct positive effects on the urinary system and is valued as a stomachic that aids weak digestion without affecting the heart. It is a drink that is mainly used as a tea substitute because it contains no harmful substances such as caffeine. It is one of the few indigenous South African plants that made the transition from the wild to a commercial product during the past 100 years. Research activities during the past 20 years have been geared towards propagation, production, genetic improvement, processing, composition, and the potential for value adding [19, 25–31, 45].

A decoction of honeybush was used as a restorative and as an expectorant in chronic catarrh and pulmonary tuberculosis [25–31, 86]. Drinking an infusion of honeybush apparently also increases the appetite, but no indication is given of the specific species [87]. According to Marloth [88], honeybush was praised by many colonists as being wholesome, valuing it as a stomachic that aids weak digestion without producing any serious stimulating effects on the heart. It also alleviates heartburn and nausea [25–31]. Anecdotal evidence suggests that it stimulates milk production in breast-feeding women and treats colic in babies [89].

Modern use of honeybush is prepared as an infusion and at times taken together with rooibos. The tea bags usually contain a larger percentage of rooibos than honeybush. Parts of other indigenous South African plants and fruits mixed with honeybush include dried buchu leaves, pieces of African potato (*Hypoxis hemerocallidea*) corms, and dried marula (*Sclerocarya birrea*) fruit. The ready-to-drink honeybush iced tea market is not developed to the same extent as that of rooibos, while honeybush “espresso,” amongst others, has not been tried [10, 19].

Honeybush is well known as caffeine-free, low tannin, aromatic herbal tea with a wealth of polyphenolic compounds associated with its health-promoting properties. The exact biologically active phytochemicals from honeybush are unknown, but the beneficial effects have been justified based on phenolic compounds. The major compounds, present in all species analyzed to date, are the xanthones, mangiferin and isomangiferin, and the flavanone, hesperidin. Recently, two benzophenone derivatives 3-*C*-*β*-glucosides of maclurin and iriflophenone were isolated for the first time from *C. genistoides* and were tested for pro-apoptotic activity toward synovial fibroblasts isolated from rheumatoid arthritis patients. The strongest proapoptotic activity was obtained for isomangiferin and iriflophenone 3-*C*-*β*-glucoside, which were not previously evaluated as potential antiarthritic agents. Proapoptotic effects were recorded for mangiferin and hesperidin, which are major polyphenols in all commercially used honeybush plants. Recently, *C. genistoides* has attracted much attention for the production of an antioxidant product high in mangiferin content. The latter and its sustainability make *C. genistoides* an attractive source of mangiferin. Other potential applications are for the prevention of skin cancer, alleviation of menopausal symptoms, and lowering of blood glucose levels [10, 19, 45, 90].

### 2.8. **Harpagophytum procumbens** (Burch.) DC. (Pedaliaceae)—Devil's Claw

*Harpagophytum procumbens* is native to the red sand areas in the Transvaal of South Africa, Botswana, and Namibia. It has spread throughout the Kalahari and Savannah desert regions. The indigenous San and Khoi peoples of Southern Africa have used Devil's Claw medicinally for centuries, if not millennia [8, 19, 91]. *Harpagophytum procumbens* has an ancient history of multiple indigenous uses and is one of the most highly commercialized indigenous traditional medicines from Africa, with bulk exports mainly to Europe where it is made into a large number of health products such as teas, tablets, capsules, and topical gels and patches [92].

Traditional uses recorded include allergies, analgesia, anorexia, antiarrhythmic, antidiabetic, antiphlogistic, antipyretic, appetite stimulant, arteriosclerosis, bitter tonic, blood diseases, boils (topical), childbirth difficulties, choleretic, diuretic, climacteric (change of life) problems, dysmenorrhea, dyspepsia, edema, fever, fibromyalgia, fibrositis, gastrointestinal disorders, headache, heartburn, indigestion, liver and gall bladder tonic, malaria, migraines, myalgia, neuralgia, nicotine poisoning, sedative, skin cancer (topical), skin ulcers (topical), sores (topical), tendonitis, urinary tract infections, and vulnerary for skin injuries. The major clinical uses for Devil's claw are as an anti-inflammatory and analgesic in joint diseases, back pain, and headache. Evidence from scientific studies in animals and humans has resulted in widespread use of standardized Devil's claw as a mild analgesic for joint pain in Europe [8, 12, 19, 91–95].

Chemical constituents according to the published literature include potentially (co)active chemical constituents: iridoid glycosides (2.2% total weight) harpagoside (0.5–1.6%), 8-p-coumaroylharpagide, 8-feruloyl-harpagide, 8-cinnamoylmyoporoside, pagoside, acteoside, isoacteoside, 6′-O-acetylacteoside, 2,6-diacetylacteoside, cinnamic acid, caffeic acid, procumbide, and procumboside. The constituent 6-acetylacteoside, being present in *H. procumbens* and absent in *H. zeyheri*, allows users to distinguish between the two species. Other compounds include flavonoids, fatty acids, aromatic acids, harpagoquinone, stigmasterol, beta-sitosterol, triterpenes, sugars (over 50%), and gum resins [8, 12, 19, 91–95]. Harpagoside isolated from *H. procumbens* varies within the plant and is the highest in the secondary tubers, with lower levels in the primary roots. The flowers, stems, and leaves appear to be devoid of active compounds. Iridoid glycosides isolated from *H. procumbens* tend to show dose-dependent anti-inflammatory and analgesic effects equivalent to phenylbutazone; they are apparently inactivated by gastric acids. Harpagoside is the most effective when given parenterally and loses potency markedly when given by mouth; enteric coated preparations might maintain efficacy despite exposure to gastric acids. Harpagoside has also been reported to inhibit arachidonic acid metabolism through both cyclooxygenase and lipoxygenase pathways [9, 91–96].

There are varying and contradictory opinions regarding the anti-inflammatory and analgesic effects of Devil's claw in the treatment of arthritic diseases and low back pain. The controversy revolves around the active constituent of Devil's claw and its mechanism of action, as it appears to be different than that of nonsteroidal anti-inflammatory drugs (NSAID). The evidence from scientific studies in animals and humans has resulted in widespread use of standardized Devil's claw as a mild analgesic for joint pain in Europe [8, 12, 19, 91–96].

Several clinical studies have been performed to determine the effectiveness of *H*. *procumbens* for its use as anti-inflammatory, general analgesic (commonly for lower back pain), and anti-rheumatic agent. To determine the effectiveness on lower back pain, *Harpagophytum* extract WS1351 was administered in two daily doses of 600 and 1200 mg containing 50 and 100 mg of harpagoside, respectively, and compared to placebo. This randomized double-blind study took place over 4 weeks and subjects (*n* = 197) with chronic susceptibility to back pain and current exacerbations with intense pain were included. Out of 183 subjects that completed the trial, six in the 600 mg and 10 in the 1200 mg were reported “pain-free” without using Tramadol (rescue pain medication). However, data analyses suggested that the 600 mg group reaped more benefits where less severe pain and no radiation or neurological deficit was present. The patients with more severe pain tended to use more Tramadol but not to the maximum permitted dose [92].

### 2.9. **Momordica charantia** Linn. (Cucurbitaceae)—Bitter Melon

*Momordica charantia*, also known as bitter melon, is a tropical vegetable grown throughout Africa. The leaf may be made into a tea called “cerassie,” and the juice, extracted from the various plant parts (fruit pulp, seeds, leaves, and whole plant), is very common folklore remedy for diabetes [2]. *M. charantia* has a long history of use as a folklore hypoglycemic agent where the plant extract has been referred to as vegetable insulin [97].

Several active compounds have been isolated from *M. charantia*, and some mechanistic studies have been done [98–101]. Khanna et al. [102] have reported the isolation from fruits, seeds, and tissue culture of seedlings, of “polypeptide-p,” a 17-amino acid, 166-residue polypeptide which did not cross-react in an immunoassay for bovine insulin. Galactose binding lectin with a molecular weight of 124,000 isolated from the seeds of *M. charantia* has been evaluated for its antilipolytic and lipogenic activities in isolated rat adipocytes and found comparable to insulin [103, 104]. Extracts of fruit pulp, seed, leaves, and whole plant of *M. charantia* have shown hypoglycemic effect in various animal models [103, 104]. Karunanayake et al. [105] found a significant improvement in glucose tolerance and hyperglycemia when the fruit juice of bitter melon was administered orally to rats. Fresh as well as freeze-dried *M. charantia* was found to improve glucose tolerance significantly in normal rats and noninsulin dependent diabetics (NIDDM) [105–108]. It was hypothesized that *M. charantia* fruit contains more than one type of hypoglycemic component. These may include an active principle called “charantin,” a homogenous mixture of *β*-sitosterol-glucoside and 2-5-stigmatadien-3-*β*-ol-glucoside that can produce a hypoglycemic effect in normal rabbits [109]. Shibib et al. [110] in a study administered the ethanolic extract of the fruit of bitter melon to STZ diabetic rats orally at a dose equivalent to 200 mg extract per kg body weight. Ninety minutes after the administration, they found that blood glucose levels had been reduced by 22%. The glucogenic enzymes-glucose-6-phosphatase and fructose 1, 6-bisphosphatase in the liver were also depressed. Further evidence for a beneficial chronic effect is that an improvement in both glucose tolerance and fasting blood glucose levels was observed in 8 NIDDM patients following 7 weeks of daily consumption of powered *M. charantia* fruit [97]. Although some authors have indicated that the effect of *M. charantia* is not associated with an increase in circulating insulin, Welihinda et al. [111] and Welihinda and Karunanayake [112] demonstrated that an aqueous extract from the fruit of *M. charantia* was a potent stimulator of insulin release from *β*-cell-rich pancreatic islets isolated from obese-hyperglycaemic mice. Recently, Matsuura et al. [113] have isolated an *α*-glucosidase inhibitor from *M. charantia* seeds which can be a potential drug therapy for postprandial hyperglycaemia (PPHG). However, Dubey et al. [104] found that the aqueous, methanolic, and saline extracts of *M. charantia* produced a significant hypoglycaemic effect in rats. In addition, the methanol and saline extracts also prevented adrenaline-induced hyperglycaemia.

When tested on laboratory animals, bitter melon has shown hypoglycaemic as well as antihyperglycaemic activities. Polypeptide-p isolated from fruit, seeds, and tissue of bitter melon showed potent hypoglycaemic effects when administered subcutaneously to gerbils, langurs, and humans. The aqueous extracts of *M. charantia* improved oral glucose tolerance test (OGTT) after 8 h in normal mice and reduced hyperglycaemia by 50% after 5 h in STZ diabetic mice. In addition, chronic oral administration of extract to normal mice for 13 days improved OGTT while no significant effect was seen on plasma insulin levels. We recently reported that *M. charantia* fruit extracts have a direct impact on transport of glucose *in vitro* [99, 114–117].

### 2.10. **Pelargonium sidoides** DC. (Geraniaceae)—Umckaloabo

*Pelargonium sidoides* is native to the coastal regions of South Africa, and available ethnobotanical information shows that the tuberous* P. sidoides* is an important traditional medicine with a rich ethnobotanical history [19].

*P. sidoides* root extract EPs 7630, also known as Umckaloabo, is a herbal remedy thought to be effective in the treatment of acute respiratory infections. There are numerous studies about *P. sidoides* and respiratory tract infections [118, 119]. These studies showed that *P. sidoides* may be effective in alleviating symptoms of acute rhinosinusitis and the common cold in adults, but doubt exists. It may be effective in relieving symptoms of acute bronchitis in adults and children and sinusitis in adults [118]. EPs 7630 significantly reduced bronchitis symptom scores in patients with acute bronchitis by day 7 [119]. No serious adverse events were reported. EPs 7630 has a positive effect on phagocytosis, oxidative burst, and intracellular killing of cells [120–125]. *P. sidoides* extract modulates the production of secretory immunoglobulin A in saliva, both interleukin-15 and interleukin-6 in serum, and interleukin-15 in the nasal mucosa [19, 126].

In one research, *P. sidoides* was documented to represent an effective treatment against common cold. It was reported to significantly reduce the severity of symptoms and shortens the duration of the common cold compared with placebo. Because of these effects, the authors have aimed at establishing whether or not* P. sidoides* could affect the asthma attack frequency after upper respiratory tract viral infection. Results for some 20 clinical trials have been published, 7 of which were observational studies and the remaining 13 were randomized, double-blind, and placebo-controlled. For 6 of those 13 trials, only preliminary results have been published. All trials have been carried out using EPs 7630 in liquid or solid forms. The data derived from these trials has been evaluated in 2 reviews [19, 118, 119].

Antibacterial activity of extracts and isolated constituents of *P. sidoides* was evaluated by Kayser [127] against three gram-positive and five gram-negative bacteria. Most compounds exhibited antibacterial activities. Further investigations by Lewu et al. [128] have supported these findings. EPs 7630 has been found to show a synergistic indirect antibacterial effect in group A-streptococci (GAS) through inhibition of bacterial adhesion to human epithelial cells as well as induction of bacterial adhesion to buccal epithelial cells [8, 129]. Wittschier et al. [130] and Beil and Kilian [131] confirmed the antiadhesive effect of EPs 7630 for *Helicobacter pylori* growth and adhesion to gastric epithelial cells. Umckaloabo has been documented to significantly stimulate phagocytosis, oxidative burst, and intracellular killing whichwas also enhanced [120–125]. It was proposed that modulation of epithelial-cell bacteria interaction through EPs 7630 may protect mucous membranes from microorganisms evading host defense mechanisms. These findings tend to provide a rationale for the treatment of upper respiratory tract infections with EPs 7630 [131, 132].

Taylor et al. [133] have recently established the antimycobacterial activity for hexane extracts of roots of *P. reniforme* and *P. sidoides*. Gödecke et al. [134] have reported no significant effect on the bacterial growth of two strains of mycobacteria by extracts and fractions of *P. sidoides*. An antitubercular effect may thus be achieved indirectly by stimulation of immune response. This assumption was supported by Mativandlela et al. [135] as none of the compounds isolated from *P. sidoides* showed any significant activity against *M. tuberculosis*.

Kayser et al. [136] have investigated into extracts and isolated constituents of *P. sidoides* for their effects on nonspecific immune functions in various bioassays. They found indirect activity, possibly through activation of macrophage functions. Activation was confirmed through the presence of tumor necrosis factor (TNF-alpha) and inorganic nitric oxides (iNO). Kolodziej [137, 138] also observed TNF-inducing potencies for EPs 7630 as well as interferon-like activities. Koch et al. [139] observed interferon- (IFN-) beta production increased and natural killer cell mediated cytotoxicity enhanced in MG-63 human osteosarcoma cells preincubated with Umckaloabo [19, 139]. Kolodziej [137, 138] investigated polyphenol-containing extracts of *P. sidoides* and simple phenols, flavan-3-ols, proanthocyanidins, and hydrolysable tannins for gene expressions (iNOS, IL-1, IL-10, IL-12, IL-18, TNF-alpha, and IFN-alpha/gamma). All extracts and compounds were capable of enhancing the iNOS and cytokine mRNA levels in parasitised cells.

## 3. Discussion

Medicinal plants are an integral part of the African healthcare system since time immemorial. Interest in traditional medicine can be explained by the fact that it is a fundamental part of the culture of the people who use it and also due to the economic challenge: on one side, the pharmaceutical drugs are not accessible to the poor and on the other side, the richness and diversity of the fauna and flora of Africa are an inexhaustible source of therapies for panoply of ailments [140]. Nonetheless, there is still a paucity of clinical evidence to show that they are effective and safe for humans. Without this information, users of traditional medicinal plants in Africa and elsewhere remain skeptical about the value of such therapies. This denies people the freedom to choose plants that are potentially less costly and are more accessible. Another issue concerning the use of botanical remedies is the need to understand the safety of these therapies. For these reasons, information about efficacy and safety of traditional medicines is urgently required. The present paper has endeavoured to overview just a few common medicinal plants from the African continent which have short- as well as long-term potential to be developed as future phytopharmaceuticals to treat and/or manage panoply of infectious and chronic conditions. Within the framework of enhancing the significance of traditional African medicinal plants, aspects such as traditional use, phytochemical composition, and *in vitro*, *in vivo,* and clinical studies pertaining to the use of these plants have been explored.

During the last few decades, it has become evident that there exists a plethora of plants with medicinal potential and it is increasingly being accepted that the African traditional medicinal plants might offer potential template molecules in the drug discovery process. Many of the plants presented here show very promising medicinal properties thus warranting further clinical investigations. Nonetheless, only few of them have robust scientific and clinical proofs and with a significant niche market (e.g., *Aloe ferox*, *Artemisia afra*, *Aspalathus linearis*, *Centella asiatica*, and *Pelargonium sidoides*) and a lot more have yet to be explored and proved before reaching the global market [7, 8, 12, 19].

In the light of modern science, significant efforts should be geared to identify and characterize the bioactive constituents from these plants. Indeed, the discovery of therapeutic compounds from traditional medicinal plant remedies remains a medically and potentially challenging task. For adventure in such an attempt, highly reproducible and robust innovative bioassays are needed in view of our limited understanding of the multifactorial pathogenicity of diseases. Innovative strategies to improve the process of plant collection are needed, especially with the legal and political issues surrounding benefit-sharing agreements. Since drug discovery from medicinal plants has traditionally been so time-consuming, it is also of uttermost importance for investigators to embark and devise new automated bioassays with special emphasis on high throughput procedures that can screen, isolate, and process data from an array of phytochemicals within shorter time lapse for product development. Additionally, these procedures should also attempt to rule out false positive hits and dereplication methods to remove nuisance compounds [7].

Nonetheless, despite continuous comprehensive and mechanism-orientated evaluation of medicinal plants from the African flora, there is still a dearth of literature coming from the last decade's investigations addressing procedures to be adopted for quality assurance, authentication, and standardization of crude plant products. Appropriate standardization could be achieved via proper management of raw material, extraction procedures, and final product formulation. Without effective quality control, consistency and market value of the herbal product may be compromised. Indeed, one of the main constraints to the growth of a modern African phytomedicine industry has also been identified as the lack of proper validation of traditional knowledge and also the lack of technical specifications and quality control standards. This makes it extremely difficult for buyers, whether national or international, to evaluate the safety and efficacy of plants and extracts, or compare batches of products from different places or from year to year. This is in marked contrast with Europe and Asia where traditional methods and formulations have been recorded and evaluated both at the local and national levels. This would also tend to justify why the level of trade of phytomedicines in Asia and Europe is blooming more than those in Africa [7].

It is also imperative that potential risk factors, for example, the contamination of medicinal plant products with heavy metals from African traditional medicine products, be addressed and that regulatory guidelines are not only carefully developed but also enforced. Controlled growth (under GACP) and processing environments (under Good Manufacturing Practice) need to ensure that contamination of medicinal plant material is kept to a minimum. For the medicinal plant industry, cultivated plant material is preferred as it is easier to control the supply chain plus contamination is nominal [141]. On the other hand, proper identification of a medicinal plant material is fundamental to the quality control process; it must be established unmistakably that the source of the plant material is genuine. Following this, microbial contamination (fungal, bacterial, and any potential human pathogens) must be checked during the stages of processing of the material. Chemical, pharmacological, and toxicological evaluations, conducted according to the principles of Good Laboratory Practices (GLPs), will certify the bioactive properties of the material undergoing processing. These tests also are often the predictors of safety of the products manufactured. Clinical safety and efficacy will need to be established through exhaustive and usually lengthy trials during the early stages of the development of a therapeutic agent. After that, so long as the standard operating procedures are adhered to, then the unit dosage forms produced will be considered safe. Notwithstanding this, quality assurance procedures must be instituted so that the products coming from the factory are of good quality, safety, and efficacy [142]. To this effect, during the development stage, product standardization, quality control and assurance, double-blind, placebo-controlled, and randomized clinical controlled trials using standardized products or products containing pure plant extracts are essential components that need to be perfected in order to translate the potential of African botanicals into a reality for human to benefit [7, 142].

## 4. Conclusions

It is evident from the literature that there is currently a renewed interest in African-plant-based medicines in the prevention and cure of various pathologies. Medicinal plants still play an important role in healthcare system in African countries. Nonetheless, there are still many major challenges that need to be overcome and addressed for its full potential to be realized as the effective treatment of diseases with plant products has not been validated thoroughly with robust scientific criteria to compete with existing conventional therapies. Additionally, other issues that need to be addressed are that of access and benefit sharing following the Nagoya agreement. Local laws need to be TRIPS compliant if trade of African herbal products is to increase, and, at the same time, issues of sustainable use and development of plant products need to be addressed.
